# Effects of systemic hypoxia on human muscular adaptations to resistance exercise training

**DOI:** 10.14814/phy2.12267

**Published:** 2015-01-19

**Authors:** Michihiro Kon, Nao Ohiwa, Akiko Honda, Takeo Matsubayashi, Tatsuaki Ikeda, Takayuki Akimoto, Yasuhiro Suzuki, Yuichi Hirano, Aaron P. Russell

**Affiliations:** Department of Sports Sciences, Japan Institute of Sports Sciences, 3‐15‐1 Nishigaoka, Kita, Tokyo, Japan; Centre for Physical Activity and Nutrition Research, School of Exercise and Nutrition Sciences, Deakin University, Burwood, Victoria, Australia; Division of Regenerative Medical Engineering, Center for Disease Biology and Integrative Medicine, Graduate School of Medicine, University of Tokyo, 7‐3‐1 Hongo, Bunkyo, Tokyo, Japan

## Abstract

This study investigated the effect of resistance exercise training performed under systemic hypoxia or normoxia on biochemical and molecular muscular adaptations in healthy male subjects. Our findings demonstrate that resistance training under systemic hypoxia led not only to muscle hypertrophy, but most interestingly, to a greater increase in muscular endurance. This increase in muscular endurance was potentially caused by the increased angiogenesis as determined by capillary‐to‐fiber ratio.

## Introduction

We are grateful for the interest that our research has attracted and are pleased to respond to the questions and comments raised. As acknowledged in the previous letter, the two important findings from our study (Kon et al. 2014) included (1) an increase in muscular endurance and, (2) the promotion of angiogenesis in skeletal muscle in subjects following resistance training under normobaric hypoxic conditions, compared to normoxia. This is the first time these adaptations had been observed following the implementation of a resistance exercise protocol traditionally used to promote hypertrophy, under normobaric hypoxic conditions. The previous letter suggested that we failed to take into consideration previously reported improvements in endurance capacity following strength‐endurance (Kon et al. 2014) and very low intensity resistance exercise training under normobaric hypoxia (Manimmanakorn et al. 2013). We respectfully disagree with this comment and aware of this work. However, these two studies implemented very different training protocols; a strength‐endurance training program of which the resistance training of 15 reps per set would suggest a low‐moderate intensity (Álvarez‐Herms et al. 2012); a very low intensity (20% 1RM) (Manimmanakorn et al. 2013) As these protocols would not be expected to increase muscle growth, but not surprisingly had a positive impact on endurance capacity, we did not believe that they were entirely relevant to our findings.

The authors suggest that for endurance athletes wanting enhanced muscular endurance performance, without an increase in body mass that comes with muscle hypertrophy, short explosive resistance training under hypoxia would be more suitable than the protocol employed in our study. We agree with the statement, but would like to add that the explosive resistance training most likely should be of a very low intensity (Manimmanakorn et al. 2013) or of a moderate intensity if combined with endurance exercise (Álvarez‐Herms et al. 2012). On the other hand, as uniquely shown in our study, recreational athletes requiring not only gains in muscular hypertrophy and strength, but also an enhancement of muscular endurance, a standard hypertrophy‐inducing resistance training protocol (70% of 1RM) performed under normobaric hypoxic conditions, would be advised.

The authors questioned whether our observations of increased angiogenesis made in recreational athletes would occur in highly trained elite athletes. This is an excellent point to raise and is an issue that concerns the majority of human exercise studies. Most studies recruit healthy recreational subjects for training and therefore are most likely to stimulate a training response, than if highly trained elite athletes were recruited. However, as a proof‐of‐concept, our observation in recreational athletes is valid. Future studies investigating the effects of a “hypertrophy‐stimulating” resistance exercise protocol performed under normobaric hypoxic conditions in elite athletes is interesting and necessary. However, in our experience it is difficult to obtain skeletal muscle samples from elite athletes.

## Conflict of Interest

None declared.
